# Quantification Bias Caused by Plasmid DNA Conformation in Quantitative Real-Time PCR Assay

**DOI:** 10.1371/journal.pone.0029101

**Published:** 2011-12-14

**Authors:** Chih-Hui Lin, Yu-Chieh Chen, Tzu-Ming Pan

**Affiliations:** Department of Biochemical Science and Technology, College of Life Science, National Taiwan University, Taipei, Taiwan; Université Paris Descartes; INSERM, U1002, France

## Abstract

Quantitative real-time PCR (qPCR) is the gold standard for the quantification of specific nucleic acid sequences. However, a serious concern has been revealed in a recent report: supercoiled plasmid standards cause significant over-estimation in qPCR quantification. In this study, we investigated the effect of plasmid DNA conformation on the quantification of DNA and the efficiency of qPCR. Our results suggest that plasmid DNA conformation has significant impact on the accuracy of absolute quantification by qPCR. DNA standard curves shifted significantly among plasmid standards with different DNA conformations. Moreover, the choice of DNA measurement method and plasmid DNA conformation may also contribute to the measurement error of DNA standard curves. Due to the multiple effects of plasmid DNA conformation on the accuracy of qPCR, efforts should be made to assure the highest consistency of plasmid standards for qPCR. Thus, we suggest that the conformation, preparation, quantification, purification, handling, and storage of standard plasmid DNA should be described and defined in the Minimum Information for Publication of Quantitative Real-Time PCR Experiments (MIQE) to assure the reproducibility and accuracy of qPCR absolute quantification.

## Introduction

Quantitative real-time polymerase chain reaction (qPCR) is the method of choice for nucleic acid sequence detection and quantification. Compared to other methods, the major advantages of qPCR are its high throughput, sensitivity, accuracy, and versatility [1]–[2]. Using DNA of known concentrations to create a calibration curve, we can quantify the precise copy number of a specific nucleic acid sequence. The DNA standards used to construct the calibration curve could be plasmid DNA, a PCR amplicon, synthesized oligonucleotide, genomic DNA, or cDNA [3]. Among these DNA standards, plasmid DNA is the most commonly used because it is relatively easy to produce and handle. Due to the quantification of absolute DNA copy number by qPCR is based on a DNA standard curve, any amplification bias or measurement error of the DNA standard will compromise the accuracy of the qPCR analysis. Since qPCR is the gold standard for DNA copy number analysis, any compromise in accuracy will be a major concern for PCR applications such as pathogen detection, food regulation, and scientific research.

It has been shown that plasmid DNA directly purified from *Escherichia coli* exists most often in supercoiled form [4]. However, the supercoiled structure of plasmid DNA is vulnerable to heat, mechanical shear, and freeze-thaw, which are common events in the laboratory. These damages could cause DNA strand breaks, changing the supercoiled plasmid into nicked-circular, closed-circular, or linear forms. Previous reports have shown that the conformation of plasmid DNA can have significant effects on DNA amplification by qPCR [5]–[7].

There are 2 major types of DNA quantification methods used in routine molecular biology experiments: UV absorbance and fluorescent dye-binding methods. UV absorbance (OD_260_) is a low-cost, moderately sensitive and reliable method to quantify high quality DNA [8]–[9]; however, the high sensitivity and specificity of fluorescent dye-binding methods have made these assays more popular in recent years.

To test the effect of plasmid DNA conformation on the accuracy of DNA quantification, we used UV absorbance as well as 2 fluorescent dye-binding methods: Hoechst 33258 dye-binding assay and Quant-iT™ dsDNA BR assay. In addition to these methods, which are used in routine molecular biology, a hydrolysis/HPLC DNA analysis method [10]–[14], which is conformation independent, was also included as a reference.

In this study, plasmid DNA samples were directly used as DNA standard after preparation to avoid DNA measurement error. The effect of plasmid DNA conformation was then investigated using 4 qPCR chemistries, including SYBR Green, TaqMan®, TaqMan® MGB (minor groove binder), and TaqMan® LNA (locked nucleic acid), by preparing DNA standard curves using supercoiled, nicked-circular, closed-circular, and linear plasmids. The consequence of amplification bias was also evaluated by PCR quantification of genetically modified (GM) maize NK603. The quantification bias caused by plasmid standard DNA conformation is of great concern in qPCR applications, which rely heavily on the accuracy of DNA quantification. Thus, the possible mechanism, consequences, and resolution of quantification bias caused by plasmid DNA conformation were investigated in this study. Our results suggest that a detailed protocol of plasmid standard DNA preparation should be followed to ensure the accuracy and reproducibility of DNA quantification and qPCR analysis.

## Materials and Methods

### Plant materials and DNA isolation

Certified reference materials (CRM) of 0.5% (ERM-BF415C), 1% (ERM-BF415D) and 5% (ERM-BF415F) GM maize NK603 content were purchased from the Institute for Reference Materials and Measurements (IRMM, Geel, Belgium). Genomic DNA was isolated from 50 mg of plant material using the GeneMark Plant Genomic DNA Purification kit (GeneMark Technolgy Co., Ltd., Tainan, Taiwan).

### Oligonucleotide primers and probes

GM maize NK603 event-specific primer set Q-NK603 F/R and Q-zSSIIb F/R was adopted from previous report [15]. Probes Q-NK603-PT, Q-NK603-PM and Q-NK603-PL were designed according to the 5′-junction sequence of GM maize NK603 (accession no. AF434709). Probes Q-zSSIIb-PT, Q-zSSIIb-PM and Q-zSSIIb-PL were designed according to the maize starch synthase gene IIb (*zSSIIb*) sequence (accession no. AF434713). Primers used in this study were synthesized by MDBio Inc. (Taipei, Taiwan). TaqMan probes and TaqMan MGB probes were designed and synthesized by Life Technologies, and the LNA TaqMan probe were designed and synthesized by Proligo LLC (Boulder Co., USA). All the sequences of primer and probes were listed in Table 1.

**Table 1:** Primers and probes used in this study.

Oligo name	Sequence (5′-3′)	Target	Amplicon size (bps)	Reference/Accession No.
Q-NK603F	CGGCCAGCAAGCCTTGTA	NK603 event specific sequence	110	(15)
Q-NK603R	CGACTCTCTTCTCAAGCATATGAATG			(15)
Q-NK603-PT^a^	**FAM**-CGGCCGCGTTAACAAGCTTACTCGA-**TAMRA**			AF434709
Q-NK603-PM^b^	**FAM**-CCGCGTTAACAAGCT-**MGBNFQ**			AF434709
Q-NK603-PL^c^	**FAM**-CGGCCG+CGTT+AACAAG+CTTACTCGA-**TAMRA**			AF434709
Q-zSSIIb-1F	CGGTGGATGCTAAGGCTGATG	zSSIIb gene	88	(15)
Q-zSSIIb-2R	AAAGGGCCAGGTTCATTATCCTC			(15)
Q-zSSIIb-PT^a^	**VIC**-TAAGGAGCACTCGCCGCCGCATCTG-**TAMRA**			AF434713
Q-zSSIIb-PM^b^	**VIC**-TAAGGAGCACTCGCCGC-**MGBNFQ**			AF434713
Q-zSSIIb-PL^c^	**FAM**-AGG+AGC+ACT+CGCCGC-**TAMRA**			AF434713

a PT: TaqMan probe.

b PM: TaqMan MGB probe.

c PL: LNA TaqMan probe, +N denotes the LNA base.

### Construction and preparation of reference plasmid samples

Supercoiled, nicked-circular closed-circular and linear form plasmid DNA standard were carefully prepared from the same batch of plasmid based on a previous report [5]. PCR products of primer set Q-NK603 F/R and Q-zSSIIb F/R were T-A cloned using yT&A Cloning Kit (Yeastern Biotech Co. Ltd., Taipei, Taiwan) to construct reference plasmids pNK and pSS for PCR assay. Reference plasmids were propagated in *Escherichia coli* JM109, and plasmid DNA was isolated using EasyPure Plasmid DNA miniprep kit (BIOMAN Scientific Co. Ltd., Taipei, Taiwan). Copy numbers of all plasmid DNA samples was estimated by Quant-iT™ dsDNA BR Assay Kit (Life Technologies Co., Carlsbad, CA, USA) before enzymatic preparations. All the plasmid samples used in this study was from the same batch. Fresh prepared plasmids were used as supercoiled form plasmid sample (S). Linear (L), close circular (C) and nicked-circular (N) form plasmids were prepared from supercoiled plasmids using restriction endonuclease SspI (NEW ENGLAND Biolabs, Inc., Ipswich, MA, USA), topoisomerase I (NEW ENGLAND Biolabs) and nicking endonuclease Nt.BspQI (NEW ENGLAND Biolabs), respectively. All the enzymatic reactions were carried with 2.5 U of corresponding enzyme at 37°C for 2 h. Plasmid DNA samples were directly used after preparation without further purification to minimize potential DNA measurement error. The integrity and the conformation of plasmid samples were confirmed using 1% agarose electrophoresis. Plasmid samples were subsequently stored at −80°C in aliquots without further purification. The effect of enzyme reaction buffer components on PCR assay was evaluated by three control samples (S-LB, S-NB and S-CB), which were prepared under the corresponding enzymatic reaction conditions without addition of enzymes.

### DNA concentration measurement

The effects of plasmid DNA conformation on DNA measurement were evaluated with three DNA quantification methods, including Quant-iT™ dsDNA BR Assay Kit, Hoechst 33258 dye-based DNA Quantitation Kit (Sigma-Aldrich, St. Louise, MO, USA) and the UV absorbance (OD_260_) method [16].

Due to the enzymatic hydrolysis process used in HPLC analysis of nucleosides, determination of DNA concentration by HPLC method has been considered as conformation and sequence-independent [10], [14]. In this study, HPLC DNA quantification method was used to provide a reference measurement which does not affect by DNA conformation. DNA samples were denatured by heating to 100°C for 2 min and held on ice for 5 min, and 25 µg of DNA were digested by nuclease P1 and alkaline phosphatase. Digestion mixture contains 5 µL of 10 mM ZnSO_4_, 10 µL of 1.0 U/mL nuclease P1 (Sigma-Aldrich), DNA sample and 30 mM sodium acetate buffer (pH 5.4) in a total volume of 100 µL. After incubating at 37°C for 16 h, 10 µL of 0.5 M Tris (pH 8.3), 6 µL of 10× NEBuffer 3 and 2 µL of 10 U/mL alkaline phosphatase (NEW ENGLAND Biolabs) were added, and the reaction mixture was further incubated at 37°C for 2 hours. HPLC analysis was performed using a chromatographic eluent pump (PU2089 plus, Jasco Co., Tokyo, Japan) with a UV detector (Jasco) set to 260 nm. Separation was done on a Luna C18 (250×4.6 mm, 5 µm) column (Phenomenex, Torrance, CA, USA) with two mobile phases- A: 50 mM KH_2_PO_4_ (pH 4.0); B: 100% methanol. The gradient program was consisted of 10 min with 0% B, followed by a linear gradient from 10 to 20 min to 20% B, followed by 20–30 min with 20% methanol. The flow rate was 1 mL/min and the injection volume was 20 µL. Calibration curves were established by the corresponding peak area of 0.02, 0.05, 0.1, 0.2 and 0.5 mM of four standard nucleosides (dA, dT, dG and dC). Sample DNA concentration was calculated according to the calibration curves.

### Effects of plasmid DNA conformation on qPCR assay

Seven groups of plasmid DNA samples (S, C, N, L, CB, NB, LB for supercoiled, close circular, nicked-circular, linear and three no-enzyme control samples, respectively) were serial diluted (from 2×10^4^ to 20 copy/µL) with deionized H_2_O according to the copy number determined by Quant-iT™ dsDNA BR Assay Kit before enzymatic preparation and used as calibration curves. PCR assay were carried out on ABI StepOne™ PCR System (Life Technologies). SYBR Green I PCR assay was carried out with SIBER Q-PCR Master Mix (BIONOVAS Biotechnology Co., Ltd. Toronto, Canada), 100 nM primer (Q-NK603F/Q-NK603R) and 1 µL plasmid DNA in a total volume of 20 µL. The PCR program was as follows: 10 min at 95°C; 40 cycles of 15 sec at 95°C and 1 min at 60°C. Melting curve analysis was carried out to assure the specificity of PCR amplification. Probe-based PCR assay including TaqMan, TaqMan MGB and LNA TaqMan, PCR reactions were carried out with TaqMan® Universal PCR Master Mix (Life Technologies), 100 nM primers (Q-NK603F/Q-NK603R), 50 nM corresponding probe (Q-NK603-PT, Q-NK603-PM or Q-NK603-PL) and 1 µL plasmid DNA in a total volume of 20 µL. The PCR program was as follows: an initial 2 min at 50°C and 10 min at 95°C, followed by 40 cycles of 15 sec at 95°C and 1 min at 60°C. PCR methods were validated by three independent runs with triplicated reactions (n = 9).

### The effect of reference plasmid DNA conformation on GM maize NK603 quantification

The effect of reference plasmid DNA conformation on GMO quantification was evaluated in this study. The GM content of GM maize NK603 CRMs (0.5%, 1% and 5% GM content) and genuine maize kernels (100%) were determined based on the supercoiled (pNK-S and pSS-S) or linear (pNK-L and pSS-L) plasmid calibration curves. Plasmid concentrations of calibration curve were 20, 50, 200, 2×10^3^, 2×10^4^ and 2×10^5^ copies. One hundred nano-gram of genomic DNA samples was used to analyze the GM content of maize sample (kernels and CRMs) with qPCR assay conditions that described in the previous part of this study. All four qPCR chemistries used in this study, including SYBR Green, TaqMan®, TaqMan® MGB and TaqMan® LNA were tested. Statistical analysis between the groups was performed using Duncan's multiple range test with SPSS Statistics software (version 17.0; IBM SPSS software, Armonk, NY, USA).

## Results

### Preparation of plasmid samples

**Figure 1:**
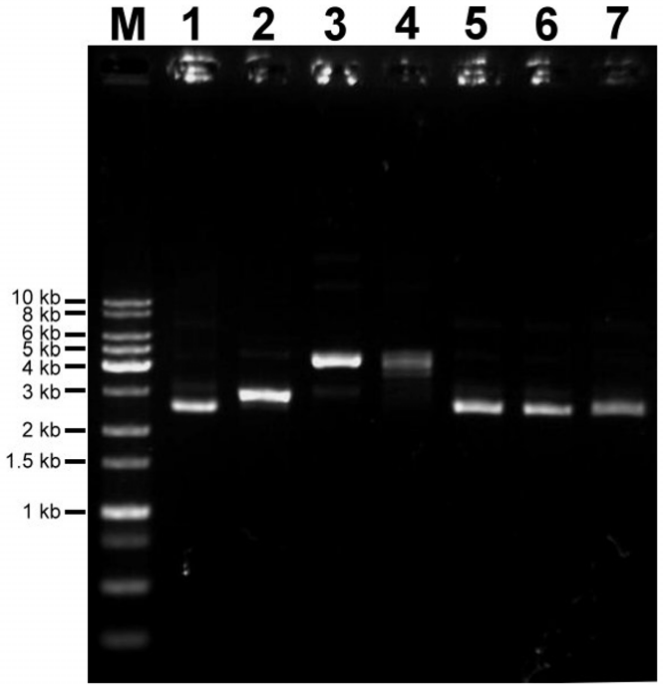
Preparation of plasmid samples. Lane M: 1 kb ladder DNA marker. Lane 1: S, supercoiled plasmid sample. Lane 2: L, linear plasmid sample (SspI treated). Lane 3: N, nicked-circular plasmid sample (Nt.BspQI treated). Lane 4: C, closed-circular plasmid sample (topoisomerase I treated). Lane 5: S-LB, supercoiled plasmid treated with SspI reaction buffer. Lane 6: S-NB, supercoiled plasmid treated with Nt.BspQI reaction buffer. Lane 7: S-CB, supercoiled plasmid treated with topoisomerase I reaction buffer.

The OD_260_ to OD_280_ ratio of supercoiled plasmid used in this study was 1.75. Linear (L), close circular (C), and nicked-circular (N) plasmids were successfully prepared from supercoiled (S) plasmids using the restriction endonuclease SspI, topoisomerase I, or the nicking endonuclease Nt.BspQI, and the integrity and conformation of samples were confirmed (Fig. 1). The results of control samples (S-LB, S-CB, and S-NB) showed that the reaction buffers and the reaction conditions used in the SspI (S-LB), Topoisomerase I (S-CB), and Nt.BspQI (S-NB) enzymes have no effect on the supercoiled conformation of plasmid DNA (Fig. 1).

### Effects of DNA conformation on measurement of plasmid DNA concentration

**Figure 2:**
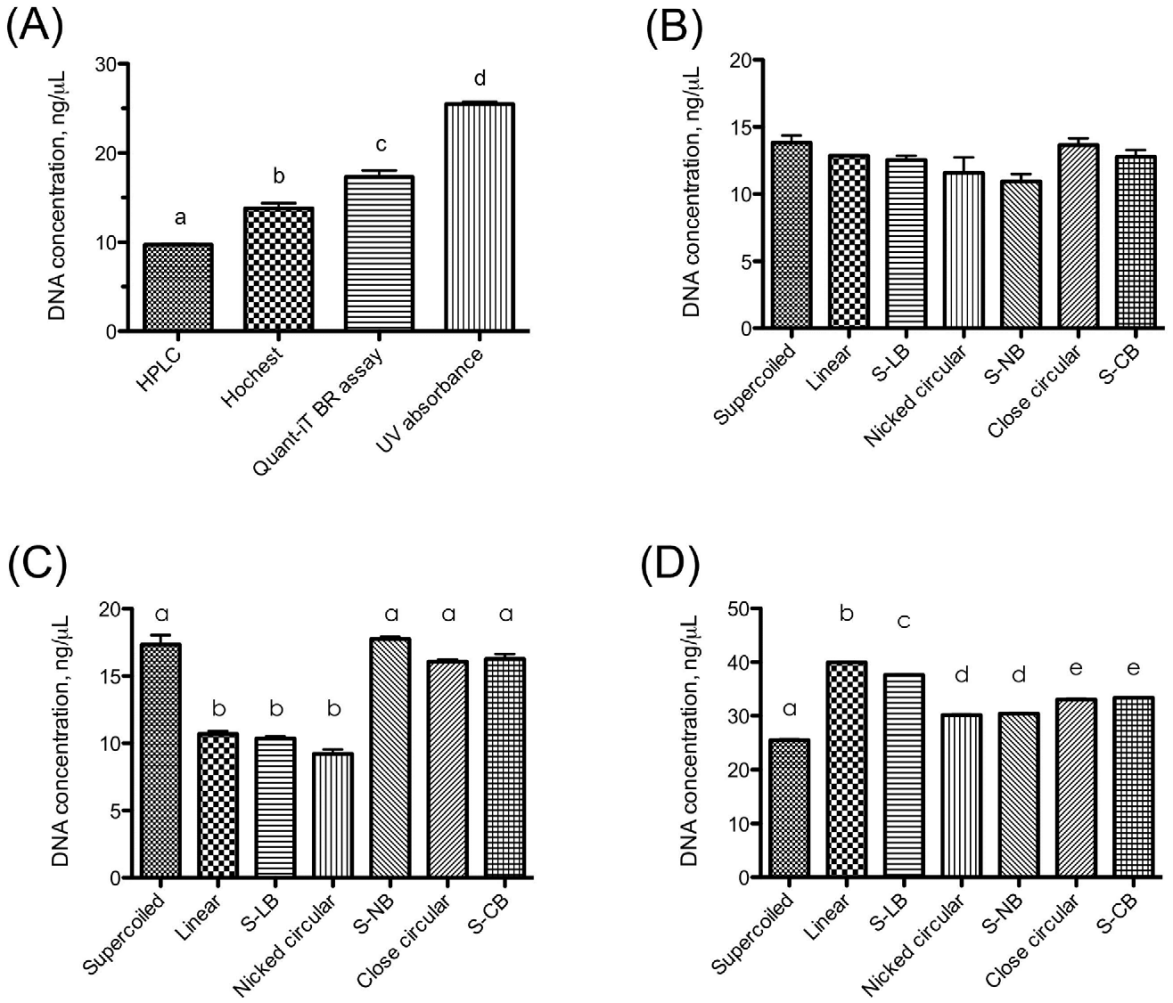
Effect of plasmid DNA conformation on DNA measurement methods. (A) Comparison of various methods on supercoiled plasmid DNA quantification. (B) Hoechst dye-based DNA quantification method. (C) Quant-iT dsDNA BR quantification assay. (D) OD_260_ DNA quantification method. S-LB: supercoiled plasmid treated with SspI reaction buffer. S-NB: supercoiled plasmid treated with Nt.BspQI reaction buffer. S-CB: supercoiled plasmid treated with topoisomerase I reaction buffer. a, b, c, d and e : groups with significant difference to each other (Duncan's multiple range test, P<0.05).

There are significant variations among the methods used to quantify supercoiled plasmid DNA in this study, with the highest variation (approximately 2.5-fold) occurring between the UV absorbance and HPLC methods (Fig. 2A). The results of DNA quantification showed that neither plasmid DNA conformation nor buffer components cause significant interference in DNA quantification by Hoechst 33258 method (Fig. 2B). However, the concentration of nicked-circular DNA was significantly lower in the Quant-iT™ dsDNA BR assay (Fig. 2C) and the concentration of linear plasmid DNA was significantly higher in the result of UV absorbance method (Fig. 2D). The results also reveal that the Quant-iT™ dsDNA BR assay is sensitive to the reaction buffer components of SspI, and the UV absorbance method is sensitive to all the reaction buffers used in this study (Fig. 2C and 2D). The highest variation caused by plasmid conformation (approximately 1.9-fold) occurs between the quantifications of supercoiled and nicked-circular plasmid DNA using the Quant-iT™ dsDNA BR assay (Fig. 2C).

### Effects of plasmid DNA conformation on qPCR assays

**Figure 3:**
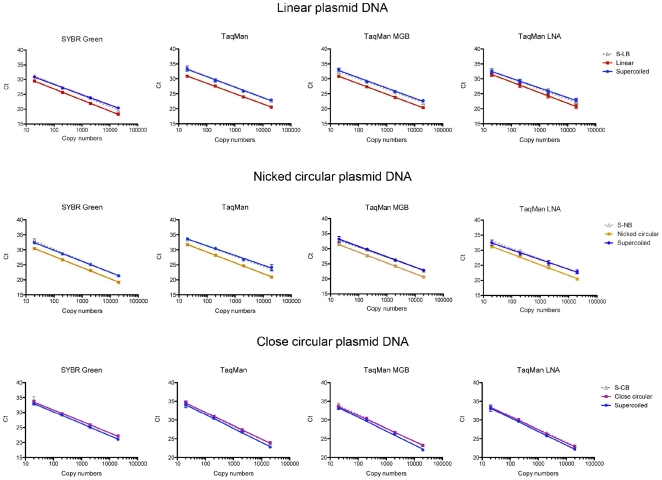
Effect of plasmid DNA conformation on qPCR calibration curves. Each data point was the average of three triplicate tests (n = 9). Blue lines: Supercoiled plasmid calibration curves. Red lines: Linear plasmid calibration curves. Gold lines: Nicked-circular plasmid calibration curves. Magenta lines: close-circular calibration curves. Grey lines: Buffer control (S-LB, S-NB and S-CB, supercoiled) plasmid calibration curves.

It has been shown that plasmid DNA directly purified from *Escherichia coli* exists most often in supercoiled form, and the supercoiled form plasmid is also the most popular form of plasmid standard DNA [4]. In this study, supercoiled, Linear (red), nicked-circular (gold), and closed-circular (magenta) plasmid DNA calibration curves were compared with the supercoiled plasmid DNA calibration curve (blue) to investigate the effect of plasmid DNA conformation on PCR chemistries (Fig. 3). The results of control samples (grey dotted line) showed that the reaction buffers and the reaction conditions of SspI (S-LB), Topoisomerase I (S-CB), and Nt.BspQI (S-NB) have no effect on the supercoiled plasmid DNA calibration curves. In this study, there was no significant difference between the PCR calibration curves of the closed-circular plasmid DNA and the supercoiled plasmid DNA. Both linear plasmid DNA and nicked-circular plasmid DNA have lower Ct values over the linear range of the PCR calibration curves (Fig. 3). Although there were minor variations on the slopes of standard curves in fig. 3, no significant effect (P<0.05) of plasmid DNA conformations on PCR efficiency was observed (Table 2).

**Table 2:** The PCR efficiency of four real-time qPCR systems on various plasmid DNA conformations.

Plasmid DNA group	PCR Efficiency (%)											
	SYBR Green I			TaqMan			TaqMan MGB			TaqMan LNA		
S	84.46	±	3.39	89.50	±	4.15	95.12	±	1.53	89.01	±	0.97
L	89.77	±	2.24	90.42	±	0.74	94.13	±	2.94	92.29	±	0.35
N	85.30	±	2.32	91.20	±	0.55	93.70	±	2.26	92.66	±	1.77
C	90.10	±	0.91	92.44	±	1.38	97.50	±	4.15	92.54	±	0.44
S-LB	89.52	±	1.39	91.63	±	7.98	95.32	±	5.60	92.61	±	4.27
S-NB	84.79	±	5.25	90.47	±	1.91	95.81	±	6.91	89.33	±	7.84
S-CB	86.14	±	2.60	91.18	±	2.73	95.75	±	1.27	92.63	±	2.19

**Figure 4:**
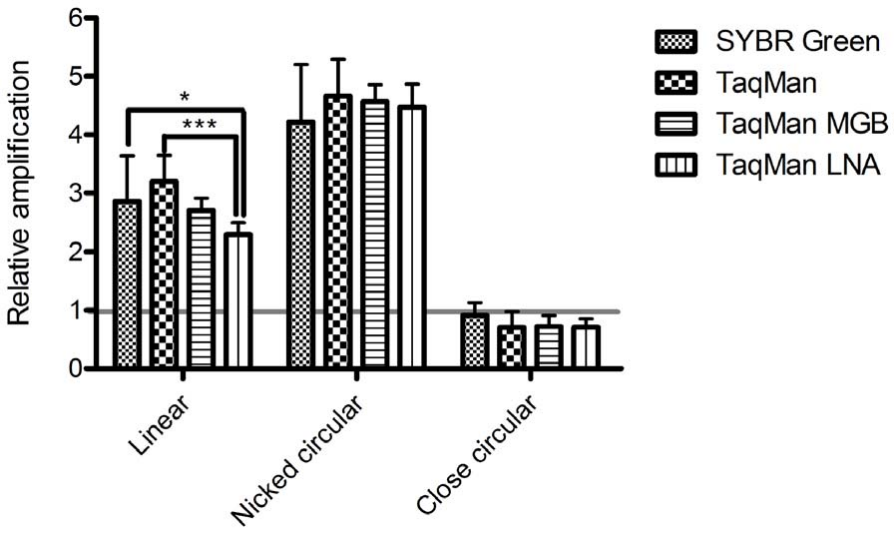
Effect of plasmid DNA conformation on qPCR chemistries. Relative amplification was calculated by the ΔCt value to the corresponding supercoiled plasmid group. Each data point was the average of three triplicate tests (n = 9) with 200 copies of plasmid DNA sample. * : P<0.05. *** : P<0.001.

The relative amplifications of 200 copies of plasmid DNA with different conformation are shown in Fig. 4. Nicked-circular plasmid DNA has the highest PCR amplification, approximately 4.5-fold greater than supercoiled plasmid DNA in all 4 PCR chemistries. The relative amplification of linear plasmid DNA is approximately 2.3- to 3.2-fold higher than that of supercoiled plasmid. Significant differences in relative amplification were observed between TaqMan LNA and TaqMan MGB, as well as between TaqMan LNA and SYBR Green chemistries (Fig. 4).

### Effects of plasmid DNA conformation on genetically modified (GM) maize NK603 quantification using PCR

In the most of routine qPCR assay, supercoiled and linear plasmid DNA were used as DNA standard. To investigate the effect of plasmid DNA conformation on genetically modified organism (GMO) quantification, certified reference material (CRM) of GM maize NK603 was analyzed based on linear and supercoiled plasmid DNA calibration curves. Plasmid DNA conformation has no significant effect on the accuracy of NK603 quantification (Table 3); however, the linear DNA calibration curve has a greater linear range than the supercoiled DNA calibration curve. This allowed for quantification of amounts of NK603 CRM that were too low to be detected using the supercoiled DNA calibration curve (Table 3 and Table 4).

**Table 3:** SYBR Green I qPCR quantification of GM maize NK603 based on supercoiled and linear plasmid DNA calibration curves.

Plasmid conformation of calibration curve	CRM level (%)	NK603-specific (copy number)			zSSIIb (copy number)			Trueness			
								Accuracy		Precision	
								Mean (%)	Bias^a^ (%)	SD	RSD^b^ (%)
Supercoiled plasmid	5.0	400.1	±	43.9	16488.2	±	2155.5	4.96	−0.77	0.12	2.37
	1.0	64.3	±	16.3	15380.1	±	901.8	0.85	−15.08	0.18	20.99
	0.5	31.9	±	7.4	11446.6	±	1365.9	0.57	13.02	0.08	14.38
Linear plasmid	5.0	106.9	±	4.9	5054.9	±	800.6	5.10	1.94	0.59	11.50
	1.0	20.6	±	2.7	5085.3	±	940.1	0.97	−2.78	0.06	5.78
	0.5	9.8^c^	±	1.9	4447.7	±	860.5	0.52	4.82	0.02	3.41

a Bias = (measured level of CRM−theoretical level of CRM)/theoretical level of CRM×100%.

b RSD: relative standard deviation = SD/Mean×100%.

c Extrapolated value.

**Table 4:** Comparison of 0.5% NK603 CRM quantifications based on suprcoiled and linear plasmid DNA calibration curves.

PCR chemistries		Plasmid DNA conformation					
		Supercoiled			Linear		
TaqMan	NK603-specific (copy number)	40.10	±	3.80	13.10^a^	±	3.10
	zSSIIb (copy number)	28612.00	±	3685.00	8421.10	±	26.10
	NK603 level (%)	0.49	±	0.05	0.46	±	0.11
TaqMan MGB	NK603-specific (copy number)	39.70	±	6.00	9.30^a^	±	1.00
	zSSIIb (copy number)	22306.10	±	33.10	7725.20	±	29.80
	NK603 level (%)	0.64	±	0.09	0.48	±	0.05
TaqMan LNA	NK603-specific (copy number)	37.00	±	4.70	10.90^a^	±	3.30
	zSSIIb (copy number)	21031.50	±	114.90	9312.50	±	38.60
	NK603 level (%)	0.59	±	0.07	0.49	±	0.14

a Extrapolated valu.

## Discussion

Quantitative real-time PCR is a powerful technique that allows direct quantification of absolute copy number of DNA without post-PCR manipulation [17]–[18]. There are 3 important variables that need to be taken into consideration when preparing a plasmid DNA calibration curve for qPCR. The first is the conformation of the DNA. It has been reported that PCR is very sensitive to conformational changes in template DNA, especially the change from supercoiled to linear [5], and that the use of supercoiled plasmid DNA standards caused serious overestimation of microalgae gene copy number [3]. The second variable is the method used to quantify the DNA. The results of this study suggest that the effect of DNA conformations to quantification methods is variable. The final variable is the qPCR method used to analyze the DNA, which is also highly variable depending on the conformation of the DNA. From this, it is clear that the generation of an accurate DNA calibration curve begins with the control of the standard plasmid DNA conformation.

The qPCR quantification bias caused by plasmid DNA conformation and its possible mechanism have been reported in previous studies [3], [5]. As the result of this study, closed-circular/supercoiled plasmids showed significant lower Ct value to nicked-circular/linear plasmids in these report. However, it is difficult to conclude these findings because different DNA measurement methods (UV absorbance and PicoGreen® dsDNA reagent) were used in these studies [3], [5]. Since the same amount of plasmid DNA in supercoiled and other conformations may have different result of DNA measurement, quantification of plasmid DNA samples after enzyme preparation will introduce DNA measurement error into the result of qPCR. Thus the DNA measurement error caused by plasmid DNA conformation may also contribute to the quantification bias in the previous studies [3], [5]. For these reasons, plasmid DNA samples were directly used as DNA standards after preparation to avoid DNA measurement error in this study.

Three common DNA measurement methods, UV absorbance, Quant-iT™ dsDNA BR assay, and Hoechst 33258 dye-binding, were tested to evaluate DNA measurement error in qPCR standard curve preparation. UV absorbance is a simple and common DNA measurement method, and with a highly purified DNA sample, the sensitivity and accuracy is satisfactory for routine molecular biology experiments. However, UV absorbance is prone to interference by impurities such as protein, RNA, and buffer components (Fig. 2D). Moreover, the comparison between measurements of linear and supercoiled plasmid DNA shows that the measurement of linear DNA is positively biased in the UV absorbance method (Fig. 2D).

Quant-iT™ dsDNA BR assay is a relatively sensitive, rapid, and low cost method to quantify DNA based on the binding of a fluorescent dye. Compared to UV absorbance, Quant-iT™ dsDNA BR assay is less susceptible to interference by buffer components (Fig. 2C). However, there is a significant negative bias observed in the measurement of nicked-circular plasmid DNA in Quant-iT™ dsDNA BR assay (Fig. 2C), which may result from the high dsDNA specificity of Quant-iT™ dsDNA BR assay. Hoechst 33258 assay is a moderately sensitive fluorescent dye-based method and is somewhat selective for dsDNA, with no significant fluorescence enhancement in the presence of RNA and proteins [19]. The Hoechst 33258 method is not affected by buffer components or plasmid DNA conformation in this study (Fig. 2B).

In addition to the 3 methods described, the enzymatic hydrolysis/HPLC method of DNA quantification was used as a reference in this study. This method is used for DNA base composition analysis [14] and is not suitable for routine DNA concentration measurement due to its time-consuming nature. However, it has been shown that the accuracy of the enzymatic hydrolysis/HPLC method is not affected by DNA conformation or by impurities [10]. The comparison of supercoiled plasmid quantification using UV absorbance, Quant-iT™ dsDNA BR assay, Hoechst 33258 dye-binding assay, and enzymatic hydrolysis/HPLC assay suggests that systematic errors occur between qPCR assays using different DNA measurement methods (Fig. 2A). Moreover, the DNA measurement bias of UV absorbance and the Quant-iT™ dsDNA BR assay suggest that plasmid DNA conformation may also be a source of error in generating a qPCR standard curve.

To examine the effect of plasmid DNA conformation on absolute quantification, a calibration curve was generated using 4 PCR chemistries. The similar Ct-shift trend of the PCR chemistries (Fig. 3) indicated that the PCR reaction, which is the common feature of all PCR chemistry, is the major source of quantification bias. Interestingly, the relative amplification of linear plasmid DNA in TaqMan® LNA chemistry was significantly different from SYBR® Green and TaqMan® chemistries (Fig. 4). This result indicates that the effect of plasmid DNA conformation might not always be consistent among qPCR chemistries.

**Figure 5:**
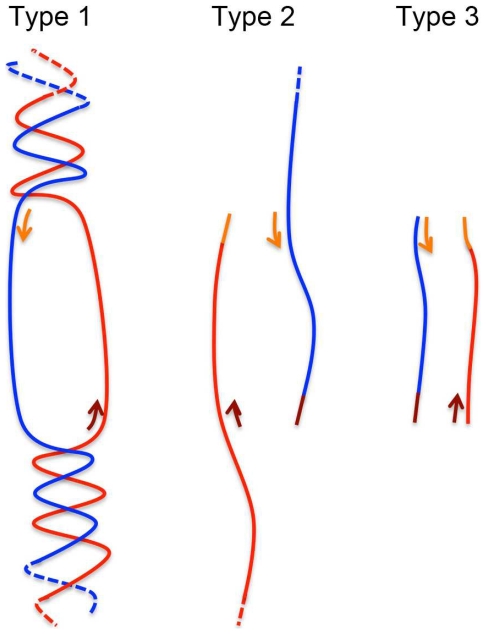
Three hypothetical type of DNA amplification in qPCR. Type I: The amplification from DNA template (plasmid). Type II : The amplification from PCR product of type I. Type III : The amplification of PCR product itself.

**Figure 6:**
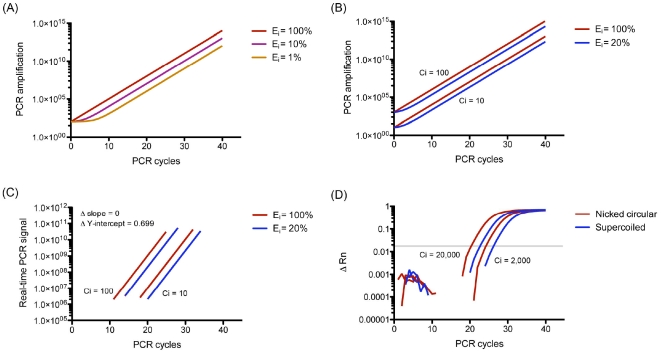
The proposed and virtual effect of initial PCR efficiency (E_i_) on qPCR amplification curves (assuming E_ii_ = E_iii_). E_i_ (initial PCR efficiency): the efficiency of type I amplification. E_ii_: the efficiency of type II amplification. E_iii_: the efficiency of type III amplification. (A) Proposed amplification curves with 100% (theoretical), 10% and 1% E_i_. (B) Proposed amplification curves of 100% and 20% E_i_ with 10 and 100 copies of DNA template (initial copy number, Ci). (C) Proposed amplification curves of 100% and 20% E_i_ with 10 and 100 Ci in PCR. Difference in E_i_ will consistently shift Ct value (by the change of Y-intercept). (D) PCR amplification curves of supercoiled and nicked-circular plasmid DNA with 2,000 and 20,000 Ci in this study.

In all 4 qPCR chemistries, the relative amplification of closed-circular and supercoiled dsDNA is significantly different than that of linear and nicked-circular DNA (Fig. 4). The major difference between closed-circular/supercoiled and nicked-circular/linear plasmids is the both strand of is a closed-circle, which causes stress (tension) to the plasmid when separate. In another word, the stress (tension) will make circular closed-circular/supercoiled plasmid DNA hard to denature in the PCR process. However, nicked-circular/linear plasmids will not have this problem since one or two strand of DNA is free to rotate. The results indicate that the PCR suppression effect of closed-circular and supercoiled plasmid is due to the tension introduced by the melting of closed-circular dsDNA, which may interfere with primer binding in PCR. However, the origin of amplification difference between linear and nicked-circular plasmids is not clear (Fig. 4). It has been reported that qPCR quantification bias between 2 genes (linear DNA) originated from the amplification efficiency of first PCR cycle [6]. In the theoretical model proposed by Nogva and Rudi [6], 3 types of amplification were occurring during the PCR process (Fig. 5): type I amplification is direct amplification of template DNA; type II amplification is the amplification of type I products; and type III amplification is the amplification of type II and type III products. If the qPCR quantification bias of closed-circular and supercoiled plasmids suppress PCR, the efficiency of type I amplification (E_i_) will be lower than type II (E_ii_) and type III amplification (E_iii_), since type II and type III DNA are linear. Table 4 shows the theoretical PCR product number with E_i._ = 20% versus E_i_ = 100%. Assuming that E_ii_ = E_iii_ = 100%, the bias will approach a constant due to the dominance of type III amplification as the PCR cycles increase (Table 5 and Fig. 6A). Thus, changes in E_i_ will cause a horizontal shift of the PCR amplification curve without modification of its slope (Fig. 6B and 6C), resulting in a constant ΔCt for each point on the calibration curve, as was observed in this study (Fig. 6D). Following this model, the supercoiled plasmid E_i_ is approximately 20% of the linear plasmid E_i_, indicating that plasmid DNA conformation has a remarkable impact on PCR amplification. It should be noted that the actual impact of plasmid DNA conformation on PCR amplification may be greater than what was observed in this study, since the denaturing step of the PCR cycle will introduce strand breaks into supercoiled plasmid DNA [5].

**Table 5:** The effect of initial PCR efficiency (E_i_ = 100% and 20%) on PCR product yield.

PCR Cycle (n)	PCR yield with 100% E_i_ (A×2^n^)	Type 1 product (A_n_)^a^	Type 2 product (B_n_)^b^	Type 3 product (C_n_)^c^	PCR yield with 20% E_i_ (D_n_)^d^	Bias^e^ (%)
0	10	10	0	0	10	0.0
1	20	10	2	0	12	−40.0
2	40	10	4	2	16	−60.0
3	80	10	6	8	24	−70.0
4	160	10	8	22	40	−75.0
5	320	10	10	52	72	−77.5
6	640	10	12	114	136	−78.8
7	1280	10	14	240	264	−79.4
8	2560	10	16	494	520	−79.7
9	5120	10	18	1004	1032	−79.8
10	10240	10	20	2026	2056	−79.9
11	20480	10	22	4072	4104	−80.0
12	40960	10	24	8166	8200	−80.0
	:				:	
	:				:	
20	10485760	10	40	2097110	2097160	−80.0
	:				:	
	:				:	
30	1.07×10^10^	10	60	2.15×10^9^	2.15×10^9^	−80.0
	:				:	
	:				:	
40	1.1×10^13^	10	80	2.2×10^12^	2.2×10^12^	−80.0

a A_n_ = A_0_ = A = initial copy number of DNA template (Ci).

b B_n_ = A×E+B_n−1_×1 (B_0_ = 0).

c C_n_ = B_n−1_×1+C_n−1_×2 (C_0_ = C_1_ = 0).

d D_n_ = A_n_+B_n_+C_n_.

e Bias = (compromised yield−theoretical yield)/theoretical yield×100%.

The absolute quantification of GMO content is an essential part of GMO regulation and the impact of plasmid DNA conformation is also demonstrated in this study. The principle of GMO absolute quantification is based on the ratio of 2 absolute quantifications. Compared to routine qPCR applications, the DNA conformation effect may be more complex since there are 2 standard plasmids (for reference and target sequence) used for qPCR quantification. In this study, the mutual offset of DNA conformation effects shows that plasmid DNA conformation will not compromise the accuracy and precision of GMO (NK603 maize) qPCR quantification if both of the standard plasmids are carefully prepared (Table 2). These results also reveal that the DNA conformation effect is independent of plasmid sequence and qPCR chemistry. Although the accuracy and precision is not affected by DNA conformation, the linear range (sensitivity) of GMO quantification is significantly changed due to the Ct shift between supercoiled and linear plasmid standard curves (Table 3 and Table 4). At 0.5% level of NK603 maize CRM, the copy number of NK603-specific sequence is undeterminable by the linear plasmid standard curve because the Ct value is out of range (<20 copies). However, by the supercoiled plasmid standard curve, the NK603-specific sequence copy number is 31.9 to 40.10 in all qPCR chemistries (Table 3 and Table 4). As a result, the sensitivity change of GMO quantification may cause significant problems for regions with a low threshold of GMO labeling requirements, such as China (0%) [20] and the European Union (0.9%) [21].

In conclusion, plasmid DNA conformation has significant impact on the accuracy of absolute quantification by qPCR. Our results suggest that the DNA conformation effect originates from the suppression of PCR efficiency by closed-circular and supercoiled plasmid DNA. It is worth noting that since the DNA conformation effect is universal for PCR, other PCR-based analysis such as quantitative competitive PCR may have the same problem. Our results also suggest that not only the plasmid DNA conformation, but also the choice of DNA measurement method, may cause significant bias when generating a DNA calibration curve. These results show that plasmid DNA conformation has multiple effects on the accuracy of absolute quantification qPCR. As a result, any compromise or change in plasmid preparation for qPCR assay may have significant impact on the accuracy and reproducibility of qPCR absolute quantification. In order to avoid these uncertainties, we suggest that the conformation, preparation, quantification, purification, handling, and storage of standard plasmid DNA should be described and defined in Minimum Information for Publication of Quantitative Real-Time PCR Experiments (MIQE) to assure the reproducibility and accuracy of qPCR absolute quantification.
